# Design optimization of cross-counter flow compact heat exchanger for energy recovery ventilator

**DOI:** 10.1007/s44189-022-00016-2

**Published:** 2022-12-21

**Authors:** Won Seok Kim

**Affiliations:** Department of Electronic, Telecommunications, Mechanical, and Automotive Engineering, Inje University, 197 Inje-Ro, Gimhae-Si, Gyeongsangnam-Do 50834 Republic of Korea

**Keywords:** Compact heat exchanger, Heat recovery ventilator, Heat transfer effectiveness, Pressure loss, Cross-counter flow

## Abstract

Energy recovery ventilators (ERVs) are the key equipment to fresh air ventilation, which is helpful for the control of respiratory diseases like COVID-19. In this paper, design optimization of the compact heat exchanger in a proposed heat recovery ventilator of the energy efficient building has been carried out and discussed. Appropriate theoretical models are required to evaluate system performance and potential energy savings. This is challenging because of the complexity of the preferred module combining cross- and counter-flow regions. The objective of the design optimization is to maximize the heat transfer effectiveness and to minimize the pressure loss of the compact heat exchanger with limited space. In this study, the allowable dimensions, heat transfer specifications and design requirements of the proposed heat exchanger are firstly defined. Then, the flow configuration, numbers, and dimensions of the air flow channels inside the heat exchanger are identified as the design parameters. A systematic design and optimization method for heat exchanger effectiveness improvement is explored. Furthermore, a detailed mathematical modeling is conducted and validated against the experimental results using the effectiveness-NTU method. It is found that the proposed modeling method is expected to be used to design of the compact heat exchanger. Finally, guidelines for improving the heat transfer effectiveness of air-to-air heat recovery ventilator were derived.

## Introduction

**Fig. 1:**
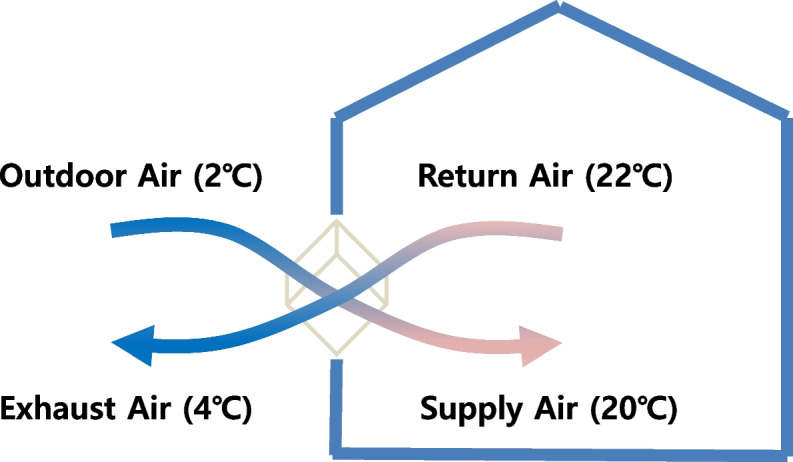
Heat recovery ventilation system

Ventilation is very important for the health and comfort of the residents. Sufficient fresh air supply is helpful for the prevention of epidemic respiratory diseases like COVID-19, but heat losses by ventilation without heat recovery are significant due to hot or cold environment outside. In order to at the same time satisfy the need for ventilation and energy saving, heat recovery means must be prepared. Recently, due to changes in awareness of the environment and the government’s strengthening of related regulations, the demand for heat exchange ventilation devices with heat recovery structure is continuously increasing, mainly in buildings and residential houses. Generally, heat recovery ventilation system means a device for exchanging heat between external fresh air and indoor warm air without installing a separate heat source (see Fig. 1). Air heat exchanger is the key equipment to fresh air ventilation system, which is helpful for energy recovery. Hence, how to promote the heat exchanger performance is commonly studied. Without heat recovery, ventilation air increases energy consumption of buildings since outdoor air must be cooled or heated to bring it close to the indoor thermal comfort conditions. Air-to-air energy recovery systems can be employed in buildings to precondition the supply air by using the exhaust air energy to reduce the energy consumption. This also reduces the size of heating and cooling facilities when the indoor air quality is satisfactory [1].

**Fig. 2:**
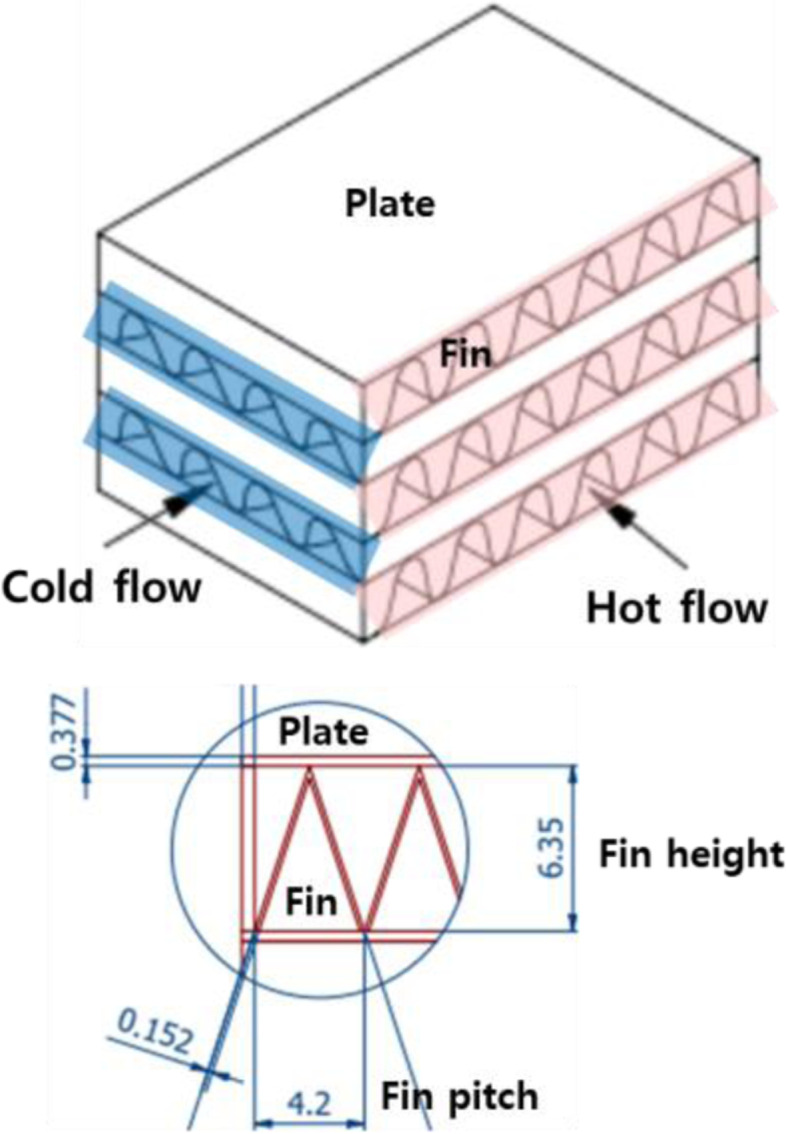
Plate-fin heat exchanger

The heat transfer coefficient of ventilator is very little due to the low temperature difference between the outside inlet air and building exhaust air and the characteristics of the sensible heat exchange between air and air. Therefore, it is required to design a high efficiency heat exchanger suitable for this. Being characterized by high heat transfer area per unit volume, compact heat exchangers are convenient for applications where space is restricted. The most common definition of compact heat exchangers in literature is heat exchangers having a surface area density greater than about 700 m^2^/m^3^. A plate fin heat exchanger (PFHE) is a type of compact heat exchanger that consists of plates and fins (see Fig. 2). The fins serve both as an extended heat transfer surface and mechanical support for the internal pressure between layers. There are many different types of fin available that make it possible to optimize the design of PFHE for any desired criterion, such as cost, weight, thermal effectiveness, or pressure drop. The design of a PFHE requires high thermal effectiveness and allowable pressure drops. Unfortunately, this type of analysis typically applies to individual fin geometry that we already know and there is no systematic method that will consider the selection of surfaces for the imposed design constraints [2].

**Fig. 3:**
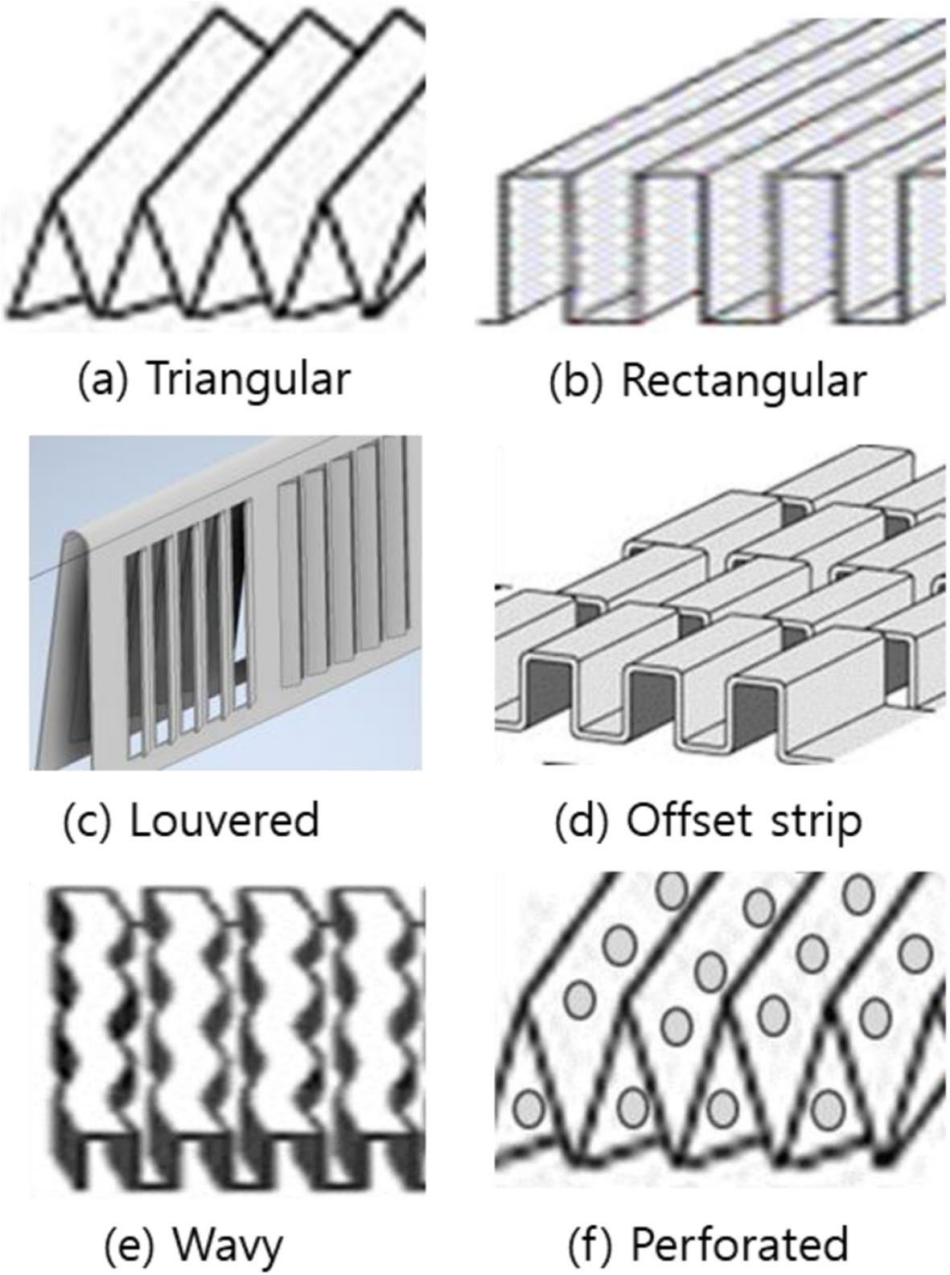
Plate-fin surface geometries

**Fig. 4:**
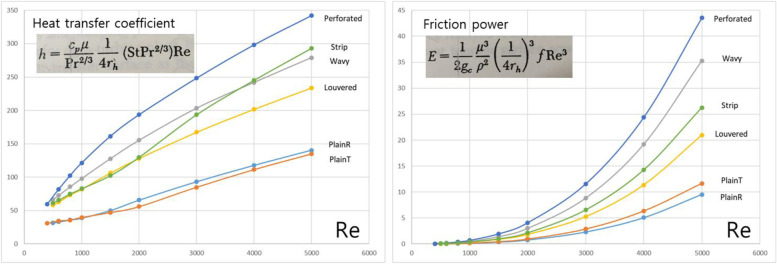
A comparison of heat transfer and friction power (recalculations using experimental data from Kays and London [2])

**Fig. 5:**
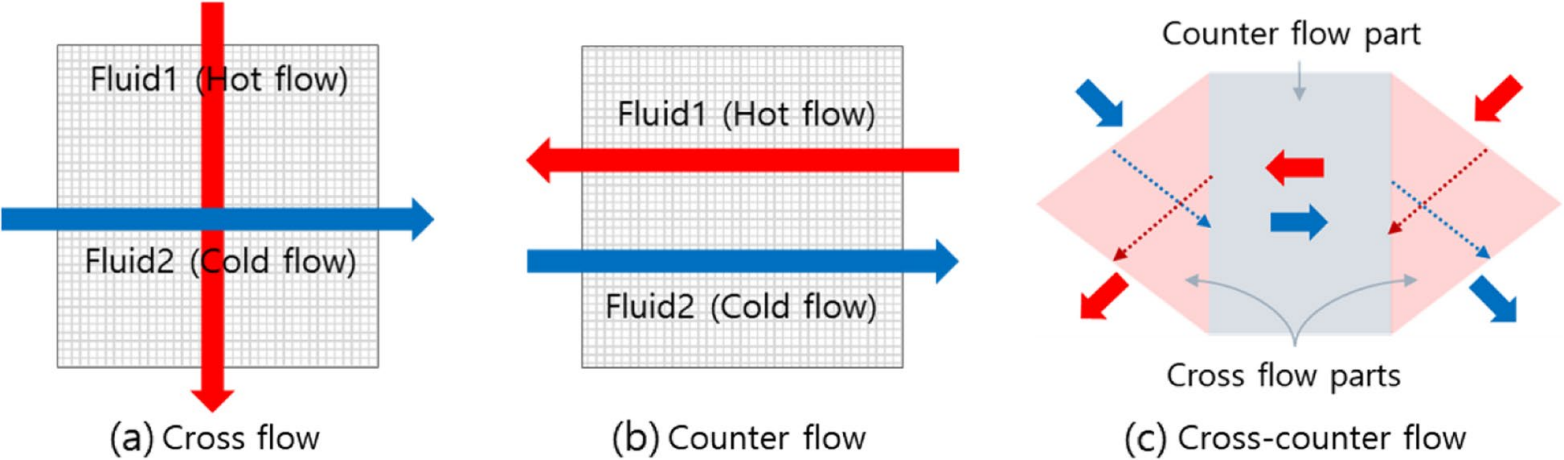
Cross, counter, and cross-counter flows

In the last two decades, significant research has been performed on investigating the compact heat exchanger performance and studying the heat transfer characteristics. The commonly used forms of the extended surface of the plate fin heat exchangers are the triangular or rectangular plain fin, offset strip fin, wavy fin, louvered fin, and perforated fin as shown in Fig. 3. For extended surface application, fin configurations fall into two categories: continuous and interrupted surfaces. Continuous surfaces achieve heat transfer enhancement through the flow patterns introduced by velocity changes, such as the triangular and rectangular fins. On the other hand, interrupted surfaces achieve heat transfer enhancement by the continuous growth and destruction of laminar boundary layers on the interrupted portion of the geometry, such as the offset strip, wavy, louvered, and perforated fins. Figure 4 shows the comparison of heat transfer coefficient and friction power of six extended surfaces on the similar compactness basis. The interesting feature of this plot is the very wide difference in heat transfer coefficient and friction power for a given Reynolds number for the continuous and interrupted surfaces. In this study, two representative fin configurations, continuous and interrupted surfaces, are carried out in each category, which are triangular and louvered fins. Those fin types are mostly used for many applications. Additionally, the calculations are separately undertaken for both cross and counter flow arrangements. In cross flow type of heat exchanger, the direction of fluid flow is normal to each other. This is one of the most common type of heat exchanger used in industrial applications and best suited for a plate fin heat exchanger because it simplifies the header design. As shown in Fig. 5a, fluid 1 and fluid 2 are flowing normal to each other and is an unmixed flow arrangement. Fluid 1 is the hot fluid which is to be cooled and fluid 2 is the cold fluid. The fins will take away the maximum heat from the fluid and transfer it to the neighboring fluid. Counter flow heat exchanger (see Fig. 5b) is the most important member of energy recovery systems. In principle counter-flow arrangement is preferred. However, other constraints should be considered when designing air-to-air compact heat exchangers. Cross flow has been the predominant flow arrangement for compact heat exchangers due to simplicity in duct sealing. Though it is simple from engineering points of view, their heat transfer effectiveness is not as high as counter-flow arrangements. As is well known, the effectiveness of a cross flow heat exchanger is generally 10% less than that of a counter flow heat exchanger, and the maximum effectiveness is limited to about mid-80%. For the counter flow, the geometry of channels and entrance cannot be directly connected such as cross flow configuration [3]. A way out is combining the advantages of cross-flow and counter-flow configurations in a single system. This yields a cross-counter flow geometry, as shown in Fig. 5c. Most studies have focused on setting cross-flows, but few have discussed cross-counter flow configurations [4]. The experimental evaluation of such heat exchangers takes a lot of time. This limits the number of parameters you can change. Counter-flow and cross-flow configurations are typically described using CFD models [5]. This is because CFD tools enable a proper simulation and helps to disclose complex flow patterns in detail. If you want to find a design tool that explains the overall module performance, the situation is different. Nasif et al. [6] have shown that experimental effectiveness of a cross-counter flow exchanger is in good agreement with numerical results obtained using CFD models. However, these CFD analyses (using such as FLUENT) have been demonstrated only for special flow systems and module geometry, depending on the tremendous computation time. Koester et al. [7] has recently shown that evaluations of system performance and potential energy conservation require an appropriate theoretical model. This is challenging since the favored module geometry combines areas of cross- and counter-flow. With CFD simulation tools it is possible to accurately discretize such geometries. However, these methods have limitations to apply to designs. To overcome such limitations, they replace complex modular geometry with a combination of standard cross-flow and counter-flow units. But they still use a commercial CFD analysis tool (Aspen Custom Modeler), meaning it is not the proper design tool. Thus, we were motivated to develop a mathematical model by substituting cross-counter flow heat exchangers with a serial connection of single cross- and counter-flow modules. In this study, first the performances with the counter-flow arrangement are compared with pure cross flow arrangements. In this analysis, the effectiveness-NTU method is an efficient yet convenient tool for performance of heat exchangers. And then a cross-counter flow heat exchanger is proposed and designed.

## Thermal–hydraulic design

### Heat transfer

**Fig. 6:**
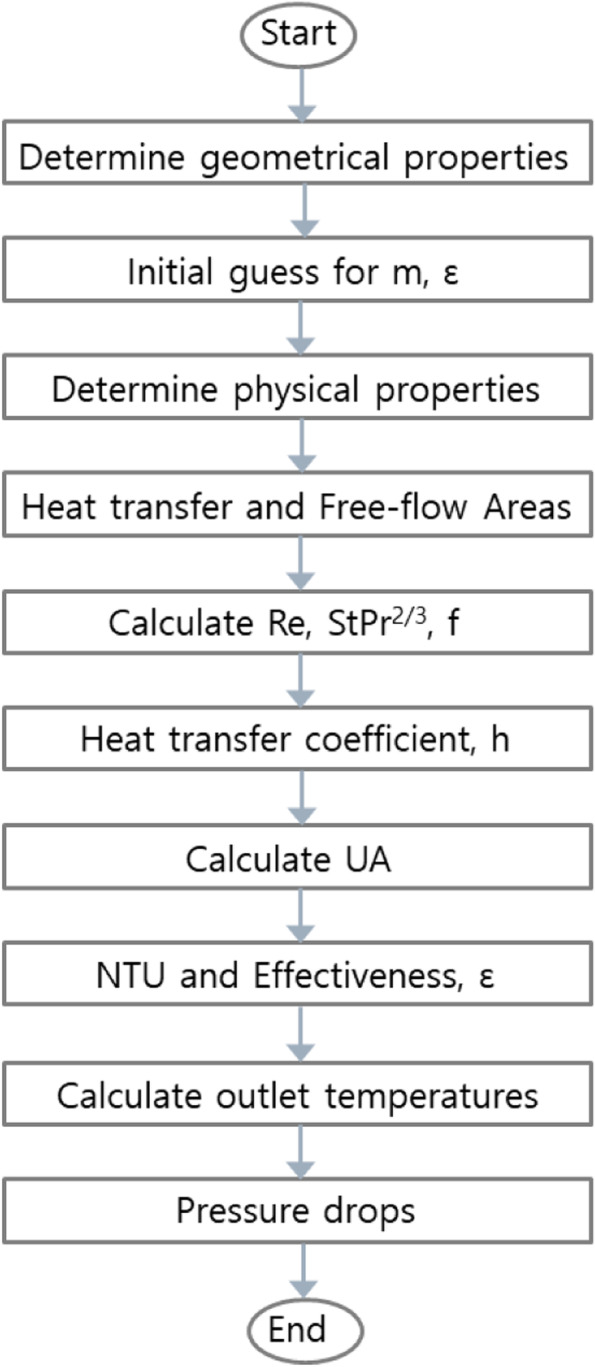
Flowchart for design of compact heat exchanger

In the thermal design of heat exchangers, it is important to determine the heat transfer and pressure drop. The design of a heat exchanger requires the specification of the heat duty, allowable pressure drops and certain aspects of heat exchanger geometry. A reasonable objective in compact heat exchanger design is the production of the smallest unit which will satisfy the required duty within the specified pressure drop constraints. Smaller heat exchanger volumes are obtained by using the surfaces that exhibit high performance. At the outset of any design exercise, the design should generally start by specifying surfaces. In other words, high performance surfaces are the first choice. The heat transfer performance of heat exchangers is usually analyzed using the logarithmic mean temperature difference (LMTD) method or the effectiveness number of transfer unit (ε-NTU) method. The LMTD method is convenient for determining the overall heat transfer coefficient based on the measured inlet and outlet fluid temperatures. On the other hand, the ε-NTU method is convenient for predicting the outlet fluid temperature when the heat transfer coefficient and inlet temperature are known. The assumptions in the development of the thermal hydraulic design for compact heat exchanger are steady state operation, single phase heat transfer process, constant fluid properties, adiabatic operation, negligible longitudinal conduction effects, uniform heat transfer coefficients and uniform flow distribution. The steps in the analysis require the determination of the following factors in the flowchart of compact heat exchanger design (see Fig. 6).

For comparative analysis, the surface geometric characteristics of the plate-fin configurations employed in compact heat exchanger are discussed as shown in Table 1. In addition, reliable correlations are derived that are essential for calculating heat transfer properties and friction coefficients. To solve the problem, inlet temperatures and mass flow rates of the heat exchanger must be determined initially. The first approximation assumes a moderate heat exchange effect and later finds a more accurate value. This approximation is used only to estimate the average temperature for fluid property assessment, so it does not have high accuracy. The exchanger heat transfer effectiveness is the ratio of the actual heat transfer rate for a heat exchanger to the maximum possible heat transfer rate, which is defined as

**Table 1:** Design specifications for comparative analysis

Surface designation	Triangular	Louvered
Plate spacing (mm)	6.35	6.35
Fin thickness (mm)	0.152	0.152
Fins/in	12	11.1
Fin pitch (mm)	2.0	2.3
Plate thickness (mm)	0.3	0.3
Hydraulic diameter (mm)	2.9	3.1
Heat transfer area/volume between plates β (m^2^/m^3^)	1288	1204

1 $$\varepsilon =\frac{q}{{q}_{max}}=\frac{{C}_{h}\left({t}_{h,in}-{t}_{h,out}\right)}{{C}_{min}\left({t}_{h,in}-{t}_{c,in}\right)}=\frac{{C}_{c}\left({t}_{c,out}-{t}_{c,in}\right)}{{C}_{min}\left({t}_{h,in}-{t}_{c,in}\right)}$$

where *C*_ℎ_ and *C*_*c*_ are the heat capacity rates of the hot and cold fluids, respectively. *C*_*min*_ is the minimum fluid capacity rate. The total heat transfer area of one side of the heat exchanger as a function of total heat exchanger volume is given by

2 $${a}_{1}=\frac{{A}_{1}}{{V}_{total}}=\frac{{b}_{1}{\beta }_{1}}{{b}_{1}+{b}_{2}+2a}$$

where α (m^2^/m^3^) is the ratio to the total surface area of one side of the heat exchanger to the total heat exchanger volume. Subscript 1 refers to any one side and 2 referred to the other side. The term *β* is the ratio of heat transfer area and volume between the plates of the heat exchanger. The total heat transfer area on each side is given by

3 $${A}_{t}=aV$$

The free flow areas are then given by

4 $${A}_{C}={\sigma A}_{fr}$$

5 $$\sigma =\frac{{A}_{c}}{{A}_{fr}}={ar}_{h}$$

where *σ* is the ratio of free flow to frontal area of one side of heat exchanger, *A*_*c*_ is free-flow area of one side, *A*_*fr*_ is frontal area of one side and *r*_*h*_ is hydraulic radius. Reynolds number is based on a hydraulic diameter and the Stanton number and friction factor are obtained from the basic characteristics of the surface. Heat transfer coefficients are calculated by the following formula:

6 $$h={StGc}_{p}$$

7 $$G=\frac{W}{A}$$

where *G* is mass flux, *W* is mass flow rate, *c*_*p*_ is specific heat. Overall coefficient of heat transfer, *U* is given by

8 $$\frac{1}{U}=\frac{1}{{\eta }_{\mathrm{0,1}}{h}_{1}}+\frac{1}{\left({A}_{t,2}/{A}_{t,1}\right){\eta }_{\mathrm{0,2}}{h}_{2}}$$

where *η*_*o1*_ and *η*_*o2*_ are surface effectiveness. If no extended surface is employed on either side, both *η*_*o1*_ and *η*_*o2*_ are unity. However, where extended surface is employed, temperature gradients along the fins extending into the fluid reduce the temperature effectiveness of the surface, and *η*_*o*_ is less than unity as a consequence. In the usual air-to-air heat exchanger, the wall resistance component may be neglected relative to the fluid side resistance. Heat transfer effectiveness can be calculated theoretically by the ε-NTU method. The number of transfer units (NTU) for the heat exchanger is defined as

9 $$NTU=\frac{UA}{{C}_{min}}$$

where *C*_min_ is equal to *C*_*c*_ or *C*_*h*_, whichever is smaller. *C*_*r*_is the heat capacity rate ratio, where *C*_min_*/C*_max_ is equal to *C*_*c*_*/C*_*h*_ or *C*_*h*_*/C*_*c*_ depending on the relative magnitudes of the hot and cold fluid heat capacity rates. The theoretical value of heat exchange effectiveness can be obtained by ε-NTU method when the value of NTU is obtained and combined with the heat capacity rate ratio.

Established ε-NTU correlations can be used for cross and counter flows of heat exchanger [3].

For the cross flow heat exchanger with both fluids unmixed (*C*_*r*_ = 1),

10 $$\varepsilon =1-exp\left[\left(\frac{1}{{C}_{r}}\right){\left(NTU\right)}^{0.22}\left\{exp\left[-{C}_{r}{\left(NTU\right)}^{0.78}\right]-1\right\}\right]$$

For the counter flow heat exchanger (*C*_*r*_ = 1),

11 $$\varepsilon =\frac{NTU}{1+NTU}$$

Therefore, the final outlet temperatures of both sides can then be computed from the definition of heat transfer effectiveness, *t*_*1,out*_ and *t*_*2,out*_ using above effectiveness value.

12 $${t}_{h,out}={t}_{h,in}-\frac{{\varepsilon C}_{min}}{{C}_{h}}\left({t}_{h,in}-{t}_{c,in}\right)$$

13 $${t}_{c,out}=\frac{{\varepsilon C}_{min}}{{C}_{c}}\left({t}_{h,in}-{t}_{c,in}\right)+{t}_{c,in}$$

### Pressure drop

In the design of air-to-air heat exchanger, the surface friction characteristics are as important as the heat transfer characteristics. The complete equation for the pressure drop is given as follows [2]:


14


where *K*_*c*_ and *K*_*e*_ are the entrance and exit pressure loss coefficients, respectively and *v*_*m*_ is the mean specific volume. The pressure drops associated with core friction, flow acceleration, entrance effect, and exit effect are considered in the correlation. In general, core friction pressure drop is the dominant term that includes almost 90% or more of the total pressure on air flow in many compact heat exchangers.

### Case study

**Fig. 7:**
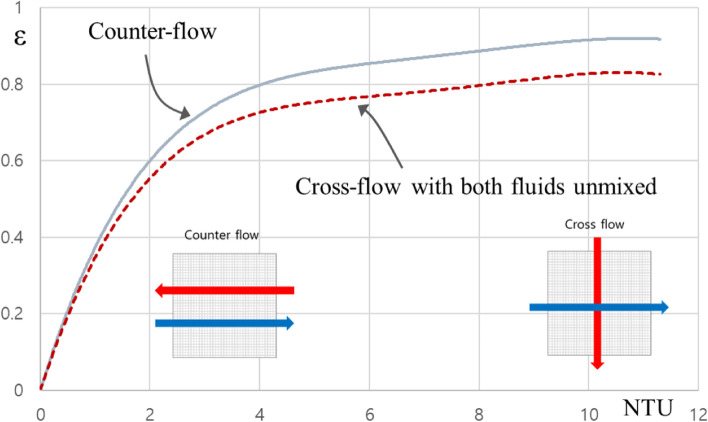
Comparison of effectiveness between cross and counter flow arrangements

Under the same thermodynamic conditions, the counter-flow arrangement of heat exchanger usually has the higher heat transfer effectiveness than the cross-flow arrangement, the reason is that the average temperature difference is higher depending on the unit length (see Fig. 7). The value of effectiveness ranges from 0 to 1. For values with small NTUs, effectiveness increases quickly with NTUs, but the higher the value, the slower. Therefore, the use of heat exchangers with large NTU cannot be economically justified, because in this case, the large increase in NTU corresponds to a small increase in the effectiveness. Thus, the heat exchanger having a very high effectiveness is very desirable in terms of heat transfer, but not be desirable in terms of economics.

**Fig. 8:**
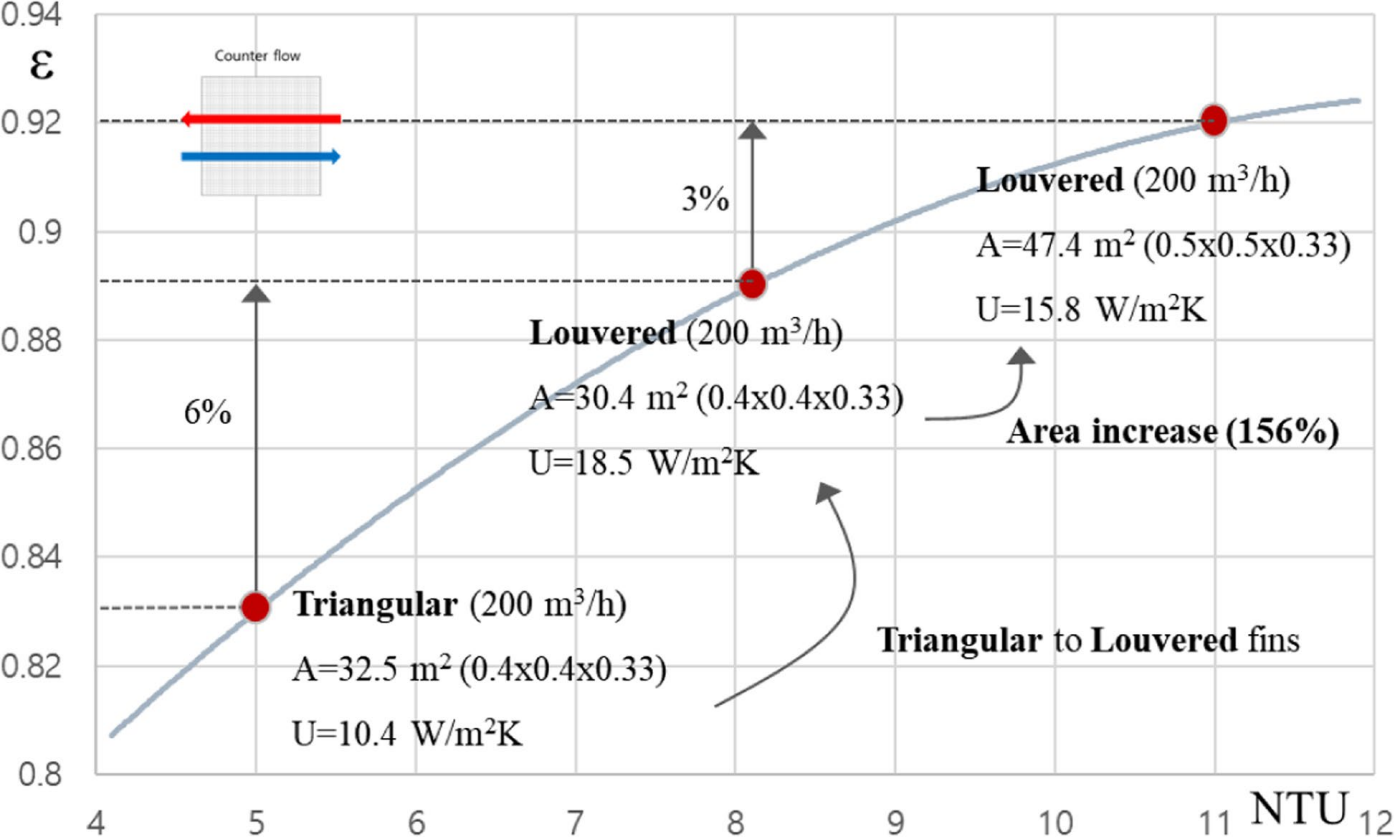
Sensitivity study of heat exchanger

Figure 8 is an analysis of the effects of using counter-flow arrangement, especially in high effectiveness area. The heat transfer of triangular and louvered fin types was compared in similar conditions, the louvered type is 6% better in terms of heat transfer effectiveness than the triangular type. In addition, the effectiveness of the heat exchanger increased by about 3% when the size of the heat exchanger was increased in the louvered type (400 × 400 × 330 → 500 × 500 × 330). As we can see from these results, it is more economical to increase the heat transfer coefficient through the change of the fin type than to increase the heat transfer area for the effectiveness improvement in the high effectiveness area (over 80%).

**Fig. 9:**
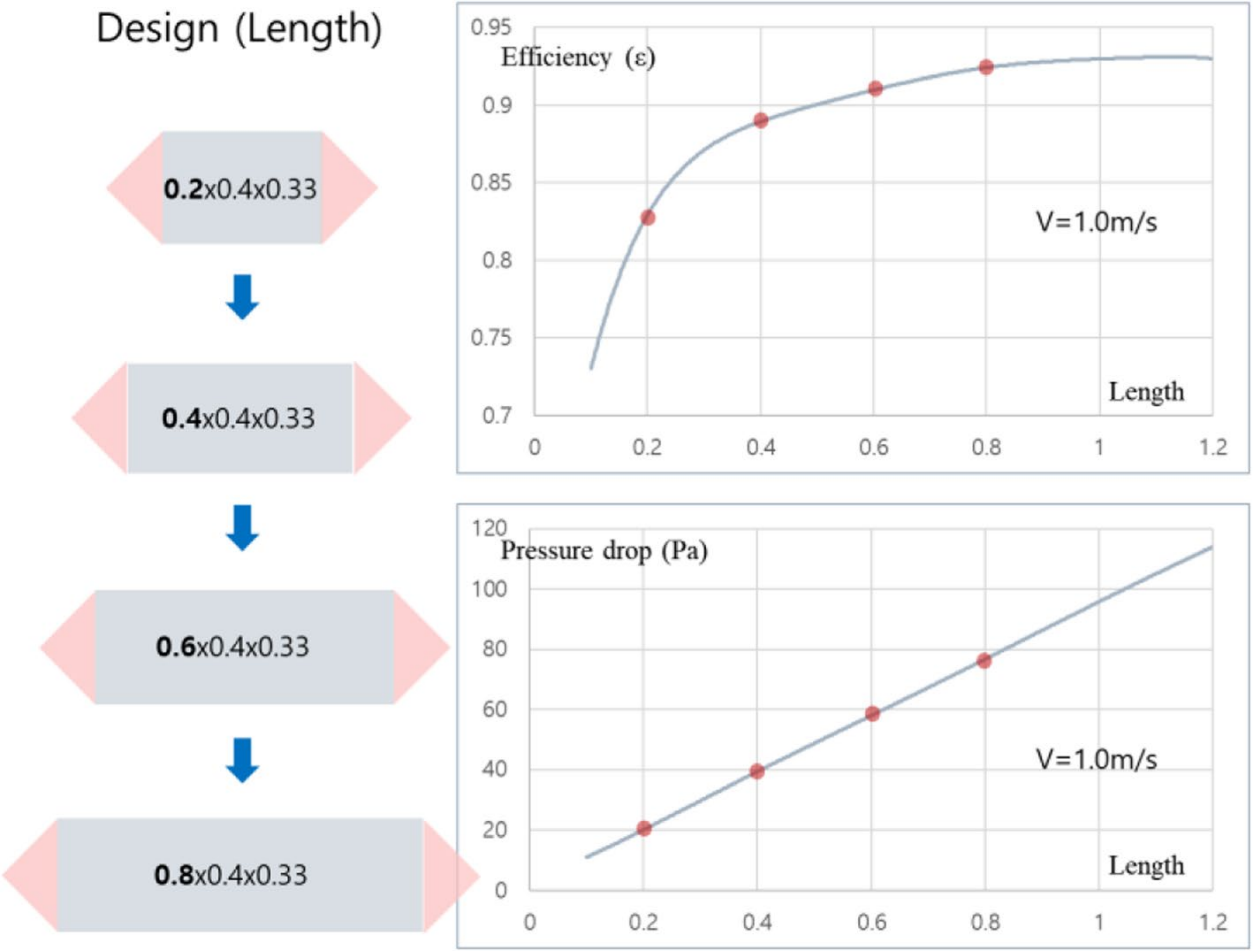
The length effect of the heat exchanger

**Fig. 10:**
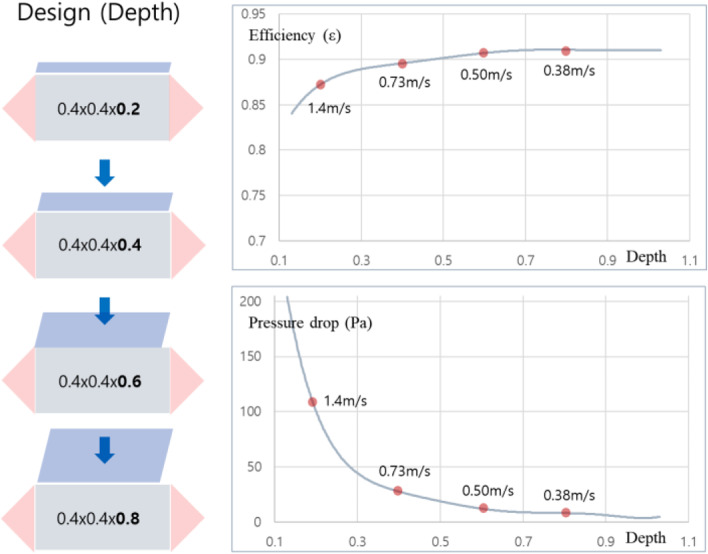
The depth effect of the heat exchanger

Figure 9 shows the sensitivity analysis of heat transfer effectiveness and pressure loss according to the length in the heat exchanger. Generally, the longer the air retention in the heat exchange element, the higher the heat exchange effectiveness. More specifically, in the first stage (length 0.1→0.4), there was an increase in effectiveness by 16%, but in the second stage (length 0.4→0.7), it was 3%, which was reduced to about 1/5 compared to the increase in effectiveness in the first stage, and there was little effect on effectiveness even if the length increased. In other words, the increase in length causes a rapid increase in effectiveness in the length of less than 0.4, but the increase in cost due to effectiveness increase is not large compared to other stages. For pressure drop, most pressure loss is caused by wall friction of the fluid, and linear increase is shown with length. Figure 10 shows the analysis of the heat transfer effectiveness and pressure drop sensitivity according to depth, and unlike the effect on the length change, the effect of the increase in depth on the effectiveness is not large, which is due to the decrease in the heat transfer coefficient by velocity despite the increase in the heat transfer area. However, in terms of pressure drop, according to the increase of depth, the pressure loss drastically diminishes in the first stage while the flow rate is dispersed. On the other hand, the slope becomes quite dull after 0.4. This also seems to require a proper compromise considering the relationship between size and pressure loss.

**Fig. 11:**
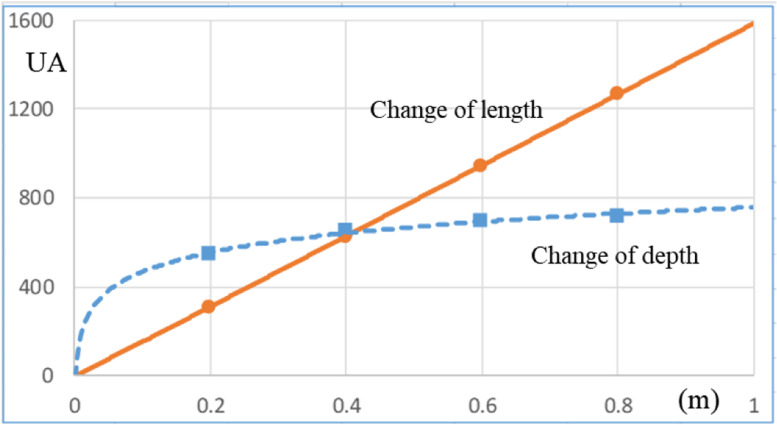
Comparison of UA at various lengths and depths

**Fig. 12:**
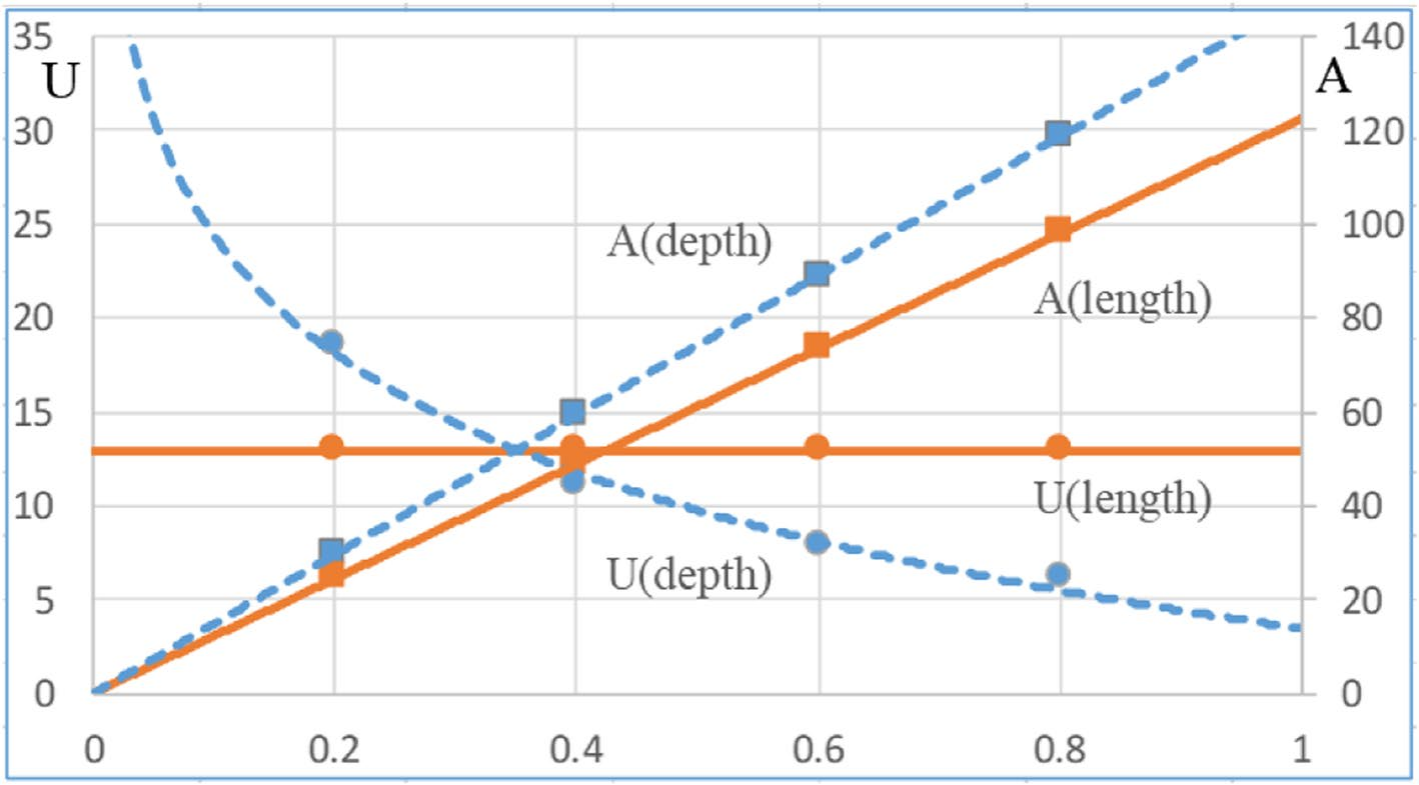
Comparison of U and A at various lengths and depths

Figure 11 shows the UA as a product of the overall heat transfer coefficient (U) and the heat transfer area (A) for the different length and depth of the counter flow heat exchanger. At fixed depth (0.33 m), as the length increases, the heat transfer coefficient does not change, while the heat transfer area increases, therefore the UA value increases linearly in proportion to the heat transfer area. On the other hand, at fixed length (0.4 m), the velocity decreases as the depth increases and the heat transfer coefficient reflecting this decreases. However, the heat transfer area increases as the depth increases, and the value of UA is offset by the values of both, showing a growth rate lower than the growth rate of the heat transfer amount (UA) due to the length change. Figure 12 shows the change of the overall heat transfer coefficient (U) and the heat transfer area (A) according to the length and depth change. Especially, in the case of depth change, the heat transfer area is larger than that at the time of length change, but the actual heat transfer (UA) is relatively small due to the decrease of the overall heat transfer coefficient.

## Results

### Design

**Fig. 13:**
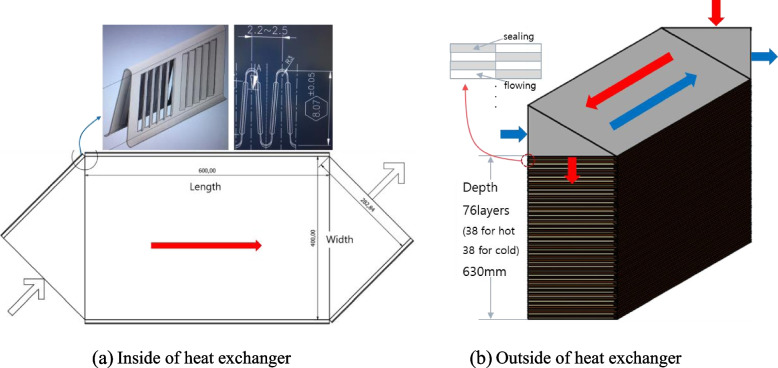
Final prototype design. **a** Inside of heat exchanger. **b** Outside of heat exchanger

**Fig. 14:**
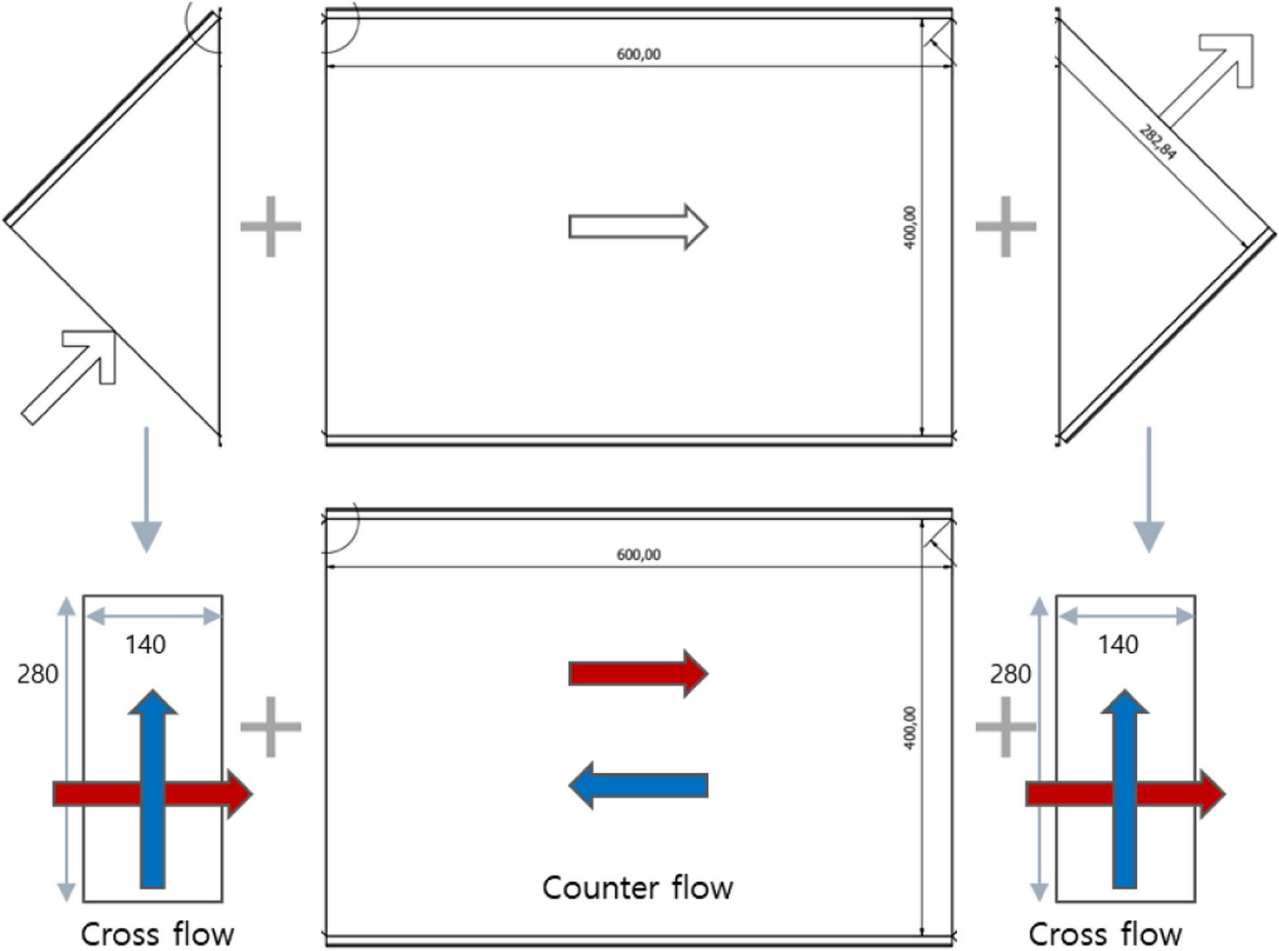
Details of the plate geometry for the design

Table 2 shows the design specifications of prototype heat exchanger. Figure 13 represents the final prototype design. The design is somewhat conservative with an effectiveness of 95% and a pressure loss of 100 Pa to meet the target of effectiveness of more than 90% and pressure loss of less than 150 Pa at 200 m^3^/h. The heat exchanger size is 600 × 400 × 630 mm and the inside flow pattern comprises the center counter-flow region and either side cross-flow region. The overall configuration is 76 layers, 8 mm in inter-layer height, 2.2 mm fin pitch, and louvered fin type. The total 76 floors are composed of 38 floors where high temperature fluid flows and 38 floors where low temperature fluid flows alternately. The material is designed with aluminum suitable for the sensible heat exchanger. For the convenience of design, this cross-counter flow heat exchanger is assumed to consist of two cross-flow modules and one counter-flow module. To properly perform the design, the triangle entrances on each side were replaced by square geometry that constituted the same surface area [4]. Details of the plate geometry for the design are shown in Fig. 14.

**Table 2:** Design specifications of prototype heat exchanger

Surface designation	Louvered
Plate spacing (mm)	8.0
Fin thickness (mm)	0.08
Fin pitch (mm)	2.2
Plate thickness (mm)	0.3
Heat transfer area/volume between plates β (m^2^/m^3^)	1916

### Manufacturing

**Fig. 15:**
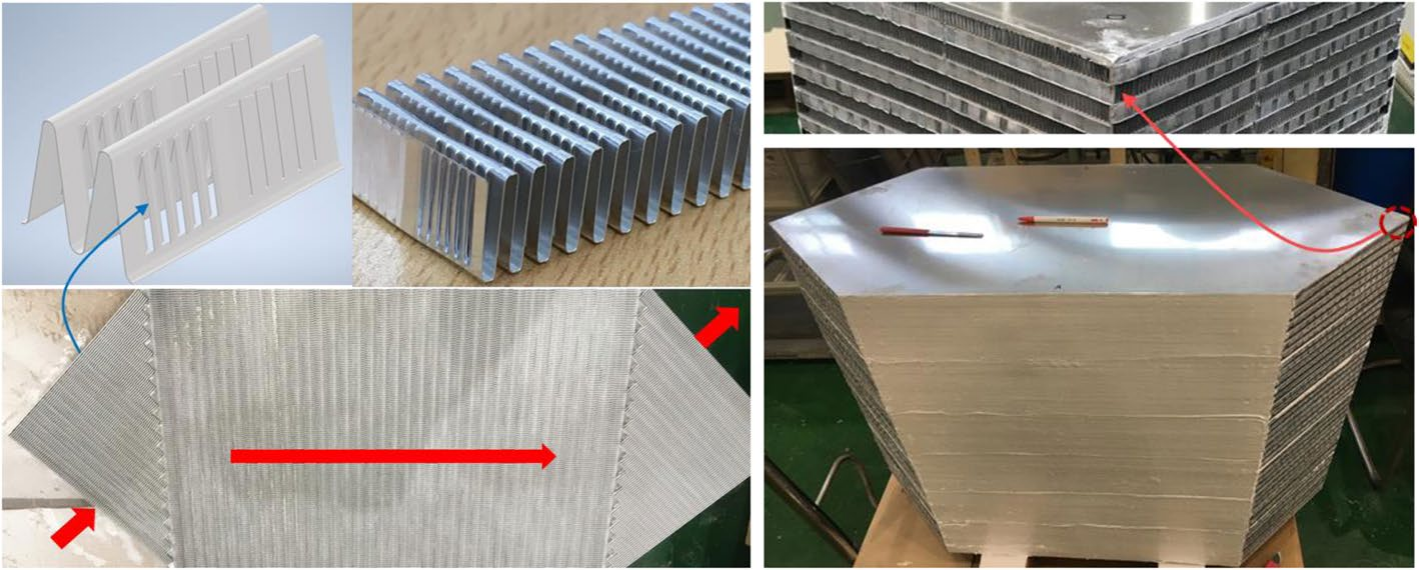
The assembly of plate-fin heat exchanger

The louvered fin is set up in each board. At this time, the hot side and cold side are distinguished and entrance and exit about these are different. The stacked plates of heat exchanger each other interlink by using the aluminum brazing method. The seal is thoroughly made so that the unnecessary leakage generate. Figure 15 shows the process of making individual plate fins, including actual louvered fins used and pre-check modeling. And also shows the entire heat exchanger, especially the corners should be carefully separated to avoid hot and cold fluid mixing.

### Performance tests

**Fig. 16:**
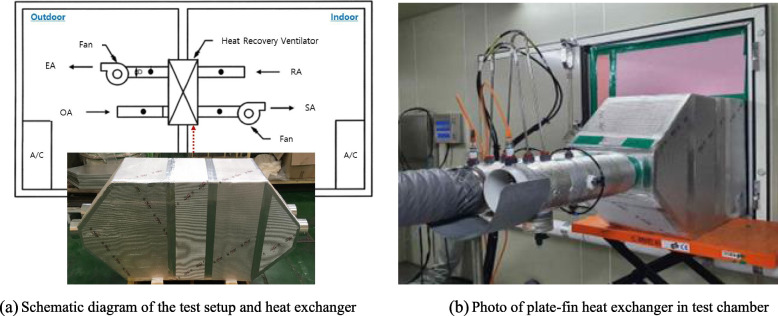
Performance test facility for plate-fin heat exchanger. **a** Schematic diagram of the test setup and heat exchanger. **b** Photo of plate-fin heat exchanger in test chamber

In this study, the environmental chamber divided into two rooms was used to confirm the performance of the heat exchanger based on KS B 6879 “Heat recovery ventilation system.” The heat exchanger and duct were insulated for accurate testing (see Fig. 16) and the test was conducted under the conditions shown in Table 3. The heat exchange effectiveness of the heat recovery ventilation system is obtained by using Eq. 15 after measuring the temperatures of outdoor air, supply air and return air.

**Table 3:** Test conditions based on KS B6879 (on heating)

Indoor temp. (℃)	Outdoor temp. (℃)
22 ± 0.3	2 ± 0.3

15 $$\varepsilon =\frac{{T}_{SA}-{T}_{OA}}{{T}_{RA}-{T}_{OA}}\times 100$$

where, ε, SA, OA, and RA are effectiveness, supply air, outdoor air, and return air (see in Fig. 16 in detail).

## Discussion

**Fig. 17:**
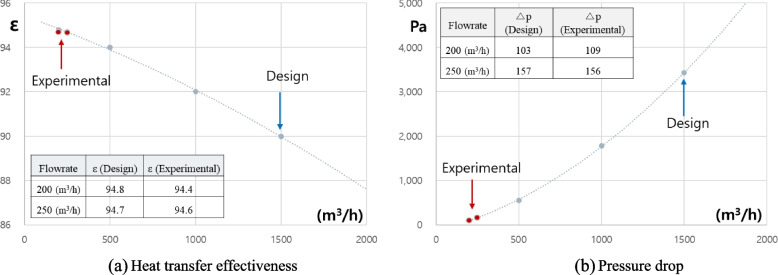
A comparative analysis of design and experiments. **a** Heat transfer effectiveness. **b** Pressure drop

In Fig. 17a, the effectiveness of the heat exchanger is shown as a function of the air flow rate. The effectiveness of the heat exchanger at 200 m^3^/h and 250 m^3^/h was 94.8% and 94.7% in design and 94.4% and 94.6% in experiment. As shown in Fig. 17a, the design predicts the experiments well. In addition, the design results show that the heat transfer effectiveness due to the increase in flow rate is decreasing. In detail, the effectiveness is determined by the ε-NTU definition as a function of NTU as shown in Eqs. 10 and 11.

16 $$NTU=\frac{UA}{{C}_{min}}\text{, where }{C}_{min}=\dot{m}{c}_{p}$$

In the related equation, NTU is a function of UA and *C*_min_, where *U* increases as flow rate increases, and *C*_min_ also increases as flow rate increases, resulting in a decrease in NTU, which leads to a decrease in effectiveness. As shown in Fig. 17b, the pressure drop increases as the flow rate increases. That is, when the flow rate increases, the pressure drop increases with the square of the velocity according to the Eq. 14.

**Fig. 18:**
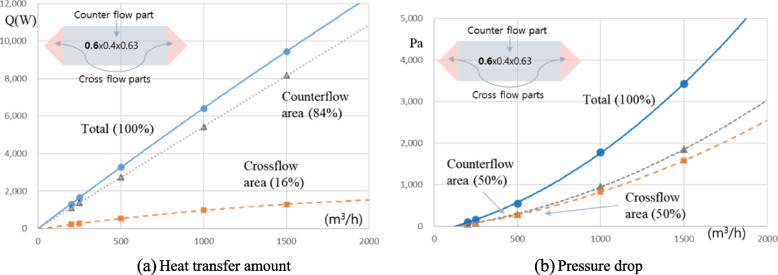
Performance comparison of the side and middle of heat exchanger. **a** Heat transfer amount. **b** Pressure drop

**Fig. 19:**
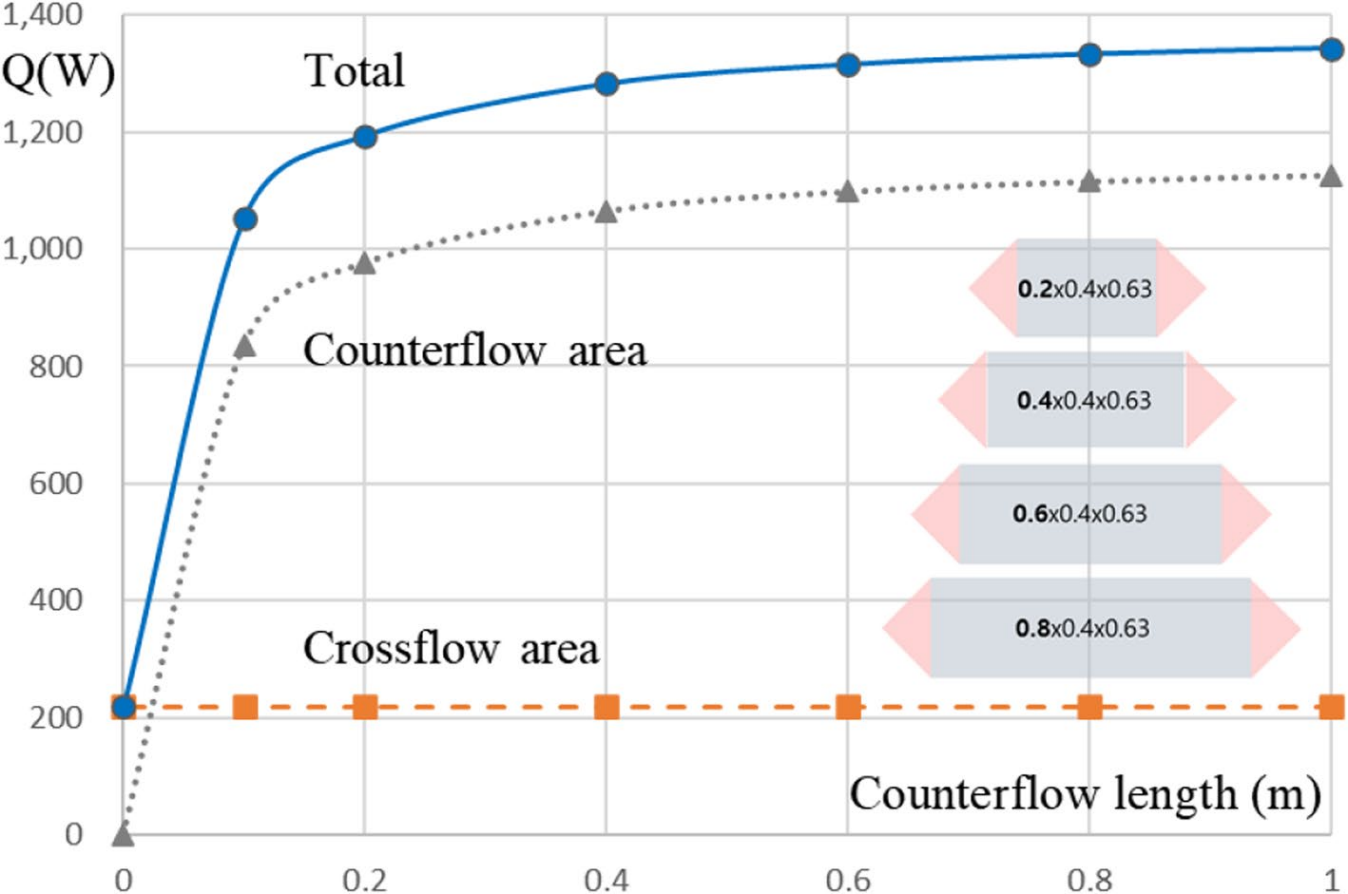
The heat transfer characteristics for the length change

Figure 18a shows the heat transfer amount at the individual domains within the heat exchanger. Most heat transfer occurs in the middle counter-flow part of the heat exchanger (about 84%) and 16% occurs in the cross-flow part of the heat exchanger side. On the other hand, Fig. 18b shows that the pressure drops for the counter-flow and cross-flow portions are 50%, respectively. The side cross-flow part of the heat exchanger is mainly used for the distribution of fluid flow and guidance to the middle counter-flow part. Figure 19 represents the heat transfer characteristics for the length change of the counter flow part by using design tool. As shown in Fig. 19, it was found that securing a minimum length of 0.2 m could have a significant heat transfer effect. Then, even if the length increases at the same rate, the heat transfer performance is significantly slowed down.

## Conclusions

The objective of the design optimization is to maximize the heat transfer effectiveness and to minimize the pressure drop of the compact heat exchanger with limited space. A systematic design and optimization method for heat exchanger effectiveness improvement is explored. Furthermore, a detailed mathematical modeling is conducted using the effectiveness-NTU method. Especially, side cross-flow and middle counter-flow parts were investigated independently. The effectiveness of the heat exchanger at 200 m^3^/h is approximately 94.8% in design and 94.4% in experiment, the design is predicting the experiment well. And also, the design results show that the heat transfer effectiveness is decreasing due to increased flow rate. On the other hand, pressure drop increases in the flow rate increase. In other words, as the flow rate increases, the pressure drop increases to the square of the velocity, because most pressure drops are caused by friction. Most heat transfer occurs in the middle counter-flow part of the heat exchanger (about 84%) and 16% occurs in the cross-flow part of the heat exchanger side. On the other hand, the pressure drops for the counter-flow and cross-flow portions are 50%, respectively. The side cross-flow part of the heat exchanger is mainly used for the distribution of fluid flow and guidance to the middle counter-flow part. Therefore, it is advantageous to design the cross flow part as small as possible. It was found that securing a minimum length of 0.2 m could have a significant heat transfer effect. Then, even if the length increases at the same rate, the heat transfer performance is significantly slowed down.

## Nomenclature

A Plate thickness

B Plate spacing

C Heat capacity rate (Wc_p_)

G Mass flux

g_c_ Proportionality factor, is equal to unity in SI unit

m Mass flow rate

p Pressure

q Heat transfer rate

t Temperature

v Specific volume

σ Ratio of free-flow area to frontal area, A_c_/A_fr_

## Data Availability

The datasets generated during and/or analyzed during the current study are available from the corresponding author on reasonable request.
